# Respiratory Syncytial Virus Prevalence and Genotypic Distribution in the Countries of the Former Soviet Union: A Systematic Review and Meta-Analysis

**DOI:** 10.3390/v18010126

**Published:** 2026-01-19

**Authors:** Denis E. Maslov, Ivan D. Osipov, Daria S. Zabelina, Anastasia A. Pak, Sergey V. Netesov

**Affiliations:** Faculty of Natural Sciences, Novosibirsk State University, Pirogov 2, Novosibirsk 630090, Russia; d.maslov8@g.nsu.ru (D.E.M.); i.osipov6@g.nsu.ru (I.D.O.); d.zabelina@g.nsu.ru (D.S.Z.); a.pak@g.nsu.ru (A.A.P.)

**Keywords:** respiratory syncytial virus, RSV, respiratory tract infections, acute respiratory infections, ARI, ARVI, *Orthopneumovirus*, systematic review and meta-analysis

## Abstract

Respiratory syncytial virus (RSV) is among leading global causes of lower respiratory tract infections, yet data from Russia and other states of the Former Soviet Union (FSU) remain fragmented and structurally inconsistent. This systematic review aims to map and synthesize existing evidence on RSV epidemiology and genotypic distribution across the FSU. Published studies from eLIBRARY and PubMed databases queried for RSV prevalence data, together with public health surveillance datasets, were used to summarize RSV prevalence research across eight FSU countries. Random-effects meta-analysis across age strata showed high prevalence in children before 6 (21%) and a progressive decline with age, which is in agreement with global data. Prevalence estimates showed a high degree of variability partially explained by study scope and clinical presentation. We observed COVID-19-related seasonal disruptions of RSV seasonality, followed by gradual post-pandemic stabilization. Genotypic data reflects global trends with two cosmopolitan clades, A.D and B.D, and their descendants, dominating in the region. The review is limited by uneven geographical and temporal coverage, and scarce data on adults. The review provides the first integrated summary of RSV epidemiology across the FSU and underscores the need for expanded regional surveillance and genomic reporting.

## 1. Introduction

Respiratory syncytial virus (RSV), or *Orthopneumovirus hominis*, is an enveloped antisense RNA virus of the *Pneumoviridae* family. Shortly after its initial discovery in chimpanzees, RSV was isolated from infants with lower respiratory tract infections (LRTIs) [1,2]. Since then, it has been recognized as one of the leading global causes of LRTI-related morbidity and mortality among children under the age of five [3]. In 2015, an estimated 33.1 million of RSV-associated acute LRTIs occurred worldwide in children, resulting in 3.2 million hospital admissions and 59,600 in-hospital deaths [4]. RSV also contributes substantially to disease burden in senior adults. In 2019, an estimated 470,000 hospitalization and 33,000 in-hospital deaths associated with RSV infection occurred in high-income countries [5]. Mortality among hospitalized seniors has been estimated at 5.6%, with a cumulative mortality rate of 25.8% within a year of admission [6]. Compared to influenza, RSV infection in this age group more frequently results in pneumonia, intensive care unit admission, and higher one-year mortality [7]. Despite this, epidemiological data in older population remain scarce.

Beyond its clinical importance, RSV imposes a considerable socioeconomic burden in many countries [8,9,10]. Historically, management of RSV infection relied almost entirely on symptomatic and supportive care, with the only targeted intervention being the monoclonal antibody palivizumab for high-risk infants. Recent advances, however, have yielded novel efficient interventions, such as long-acting monoclonal antibodies like nirsevimab and vaccines approved for senior adults and pregnant women [2,11]. While RSV’s burden is widely recognized, these interventions are predominantly deployed in high-income countries, and their adoption lags significantly in other regions, including the Former Soviet Union (FSU), where the means and models for implementation have not kept pace.

Following the dissolution of the USSR, successor states faced a rise in child mortality from respiratory infections and tuberculosis, extending the patterns already evident in the late Soviet period [12]. Although child mortality had markedly declined by the late 2010s, significant gaps in healthcare capacity persist, especially in rural areas and in lower-income countries of the region [13]. The FSU region spans countries ranging from high-income economies, such as Baltic States, to lower-middle-income countries in Central Asia. Most fall within the upper-middle-income group by World Bank Group classification [14]. This diversity leaves the region misaligned with the usual contrasts used in global health research, separating high-income settings from low-middle-income ones. Consequently, Russia and other FSU countries remain underrepresented in the global epidemiological literature, and the available data are often fragmented and structurally inconsistent. No dedicated synthesis of RSV prevalence across the FSU has been published to date, and previous global systematic reviews [3,15,16,17] have included limited information from Russia and neighboring post-Soviet countries, often aggregated within broader regional estimates. This circumstance is compounded by the poor accessibility of studies produced by research collectives in the region, as many are rarely translated to English and unindexed in international databases. As a consequence, the epidemiological landscape of Russia and countries of the FSU remains largely uncharted.

This systematic review, therefore, aims to map and synthesize existing evidence on RSV epidemiology in the FSU countries, focusing on prevalence estimates. We also analyzed broad RSV seasonality based on cross-regional research and sentinel surveillance programs and summarized viral subtype and clade distribution. By bringing the available data together, this review outlines the current state of knowledge on RSV circulation in the region.

## 2. Materials and Methods

The protocol for this systematic review/meta-analysis was retrospectively registered with the Open Scientific Framework (OSF, https://osf.io/ph4m2 (accessed on 11 December 2025)) [18] and reported in accordance with the Preferred Reporting Items for Systematic Reviews and Meta-Analyses (PRISMA) guidelines 2020 [19].

### 2.1. Definitions

The prevalence of RSV was defined as the proportion of positive-tested subjects among all individuals tested for this virus within a given group. The main outcome of our analysis is the RSV prevalence. We analyzed prevalence across different geographic regions, seasons, and categories of clinical presentation. The secondary outcome is distribution of RSV subtypes, genotypes, and clades.

Throughout the manuscript, the terms “study” and “record” refer to a source of data, a published paper, conference proceedings, or independent dataset. The term “entry” denotes an analytical unit within a study or record corresponding to a distinct age group, region, time period, etc.

Geographic regions within the Russian Federation were classified according to the official administrative division into federal districts. For other post-Soviet countries, regional attribution was made at the country level, e.g., any study conducted within the Republic of Belarus was designated as “Belarus”. For the purposes of temporal distribution analysis and clarity of presentation, several countries were grouped under broader categories: Georgia and Armenia under “Transcaucasia”; Kazakhstan, Uzbekistan, Turkmenistan, and Kyrgyzstan under “Central Asia”; Estonia, Latvia, and Lithuania under “Baltic States”.

Patient data were stratified by age into the following groups: (a) infants (<1 year); (b) toddlers (1–2 years); (c) preschoolers (3–5 years); (d) school-age children (6–17 years); (e) adults (18–64 years); and (f) senior adults (>64 years). When the age group reported in the original study overlapped with two strata, it was assigned to the most appropriate stratum based on reported age distribution, or assigned to the strata it has greater coverage of. Such cases were flagged in the “age_bridge” column as TRUE to indicate bridging. Age groups overlapping equally across two strata or spanning multiple strata were omitted from the analysis and assigned to either “Broad children” (<18 years) stratum, or to the “Mixed” group, with the latter also covering entries explicitly reporting prevalence over diverse or unspecified age groups.

To facilitate structured analysis of heterogenous clinical presentations, data were stratified into three distinct clinical categories based on diagnostic descriptions provided by original authors: ARI (acute respiratory infection), LRTI (lower respiratory tract infection), and pneumonia. This approach acknowledged the variability in diagnostic practices across studies and relied on clinical classifications according to descriptions provided in primary publications, as most studies did not specify adherence to particular diagnostic guidelines (e.g., WHO or ECDC case definitions). Cases were classified as ARI if cohorts were described as patients presenting with symptoms or diagnosis of acute respiratory infection, acute respiratory viral infection (ARVI), influenza-like illness (ILI), or severe acute respiratory infection (SARI) without explicit mention of lower respiratory tract involvement. The LRTI group encompassed cohorts described as having an LRTI or specific clinical diagnosis characteristic of LRTI such as bronchiolitis, acute bronchiolitis, obstructive bronchiolitis, or laryngobronchiolitis. The pneumonia group was reserved exclusively for cohorts with radiologically confirmed pneumonia.

### 2.2. Search Strategy/Selection Criteria

On 31 August 2025, eLIBRARY, the most comprehensive database of scientific works in the Russian language (https://elibrary.ru/defaultx.asp (accessed on 11 December 2025)), was searched using keywords referring to RSV (“pecпиpaтopный” and “cинцитиaльный”). Terms associated with bovine RSV, model, and therapeutics study were excluded (see Supplementary Table S1 for exact search queries). Given that the eLIBRARY database primarily indexes publications in the Russian language, no geographic filters were applied. On 13 September 2025, a complementary search was conducted in PubMed, a biomedical literature database of the National Library of Medicine (https://pubmed.ncbi.nlm.nih.gov (accessed on 11 December 2025)). The search terms were related to (a) respiratory syncytial virus, (b) epidemiology and prevalence, and (c) studies conducted within the FSU or USSR. No date or language filters were used in either of the databases.

After removal of duplicates and of Russian language records not indexed by Russian Scientific Citation Index, D.E.M. and I.D.O. independently screened all retrieved titles and abstracts for eligibility. In case of disagreement, the record was retained in the shortlist compiled as a result of abstract/title-based screening. The full texts in the shortlist were further assessed for eligibility independently by D.E.M., I.D.O., and D.S.Z., adhering to the same exclusion/inclusion criteria. During full-text screening, the corpus was randomly split in roughly equal parts to ensure at least twofold independent coverage of each record. Disagreements were resolved by discussion between two reviewers; if consensus was not reached, the record and the arguments of the two reviewers were referred to a third reviewer who had not been involved in the review of this specific source. The third reviewer made the final decision on inclusion.

**Figure 1:**
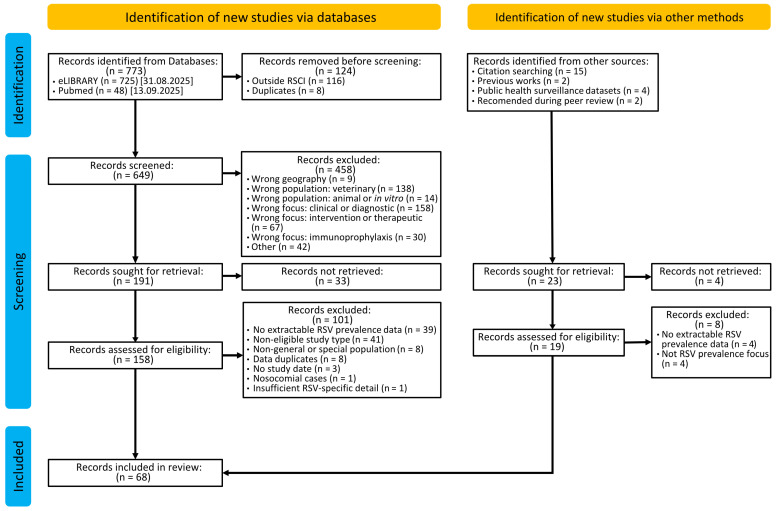
The PRISMA 2020 flowchart summarizing study selection process. The diagram reflects the flow of records through the identification, screening for eligibility, and inclusion stages of the systematic review. Numbers of records retrieved from eLIBRARY and PubMed databases, duplicates removed, title/abstract exclusions, full-text assessments, and final studies included in the analysis are shown. Additional studies identified via citation searching, publicly available surveillance data, and recommended during peer review are displayed under “Identification of new studies via other methods”.

Outside the traditional database search, additional studies identified via citation searching, among relevant works already known to the authors (“Previous works” in Figure 1), and two sources recommended during peer review were included in the shortlist and screened using the same procedure outlined above. In addition to published studies, publicly available surveillance data from the Russian Federation [20], Kazakhstan [21], and the WHO FluNet platform [22] were included as additional sources for RSV prevalence estimates. Data retrieved from the FluNet platform were limited to sentinel surveillance reports; FluNet data for Russia and Kazakhstan were not used, to avoid overlap with the national datasets. Weekly national influenza and ARVI bulletin from Uzbekistan [23] was considered as a potential additional data source, but was not included because RSV prevalence could not be reliably extracted.

Inclusion criteria required that studies be written in Russian or English, originate from a country of the FSU or USSR, and met one of the following conditions: report RSV prevalence for at least 50 individuals (with numerator and denominator extractable or inferable), or provide data on RSV subtype, genotype, or clade distribution. Full PICOS inclusion/exclusion criteria are detailed in Supplementary Table S2. The study selection process is summarized in Figure 1. The complete list of included records appears in Appendix A Table A1; entries from records reporting RSV prevalences are provided in Supplementary Table S3, and those reporting genotypic data are provided in Supplementary Table S4.

### 2.3. Data Extraction/Bias Assessment

Prior to data extraction, all studies were screened for possible data duplication. Study regions, authors, and participant characteristics were compared, and when substantial overlap could not be conclusively excluded, only one record was retained. When overlap could be identified and accounted for, the records were kept but treated as a single analytic unit during synthesis. Details on suspected data duplications and exclusion decisions are provided in Supplementary Table S5.

Data extraction from all included studies was performed independently by D.E.M., I.D.O., and D.S.Z., and then compared and adjusted in cases of disagreements. The following was included in the summary: title in the original language, authors, journal, publication year, doi, pmid, and eLIBRARY identifier, whether the study targeted only RSV or other pathogens as well, co-detection status, study location and time period, number of RSV-positive participants and total number tested, prevalence, age group, population type (inpatient, outpatient, or mixed, community, or special population), and reported clinical presentation. The summary table was arranged so that entries corresponding to a single study had zero sample overlap. The only exception was co-detection data, which were presented alongside the marginal prevalences and explicitly marked as co-infections.

Risk of bias was assessed independently by I.D.O., D.S.Z., and A.A.P. As no existing tool fully captured the features relevant to the compiled RSV prevalence dataset and the regional publishing specifics, we developed a custom critical-appraisal checklist. Its structure is based on recurring methodological limitations observed during screening and on the principles for prevalence studies appraisal described in Chapter 5 of Joanna Briggs Institute Reviewer’s Manual [24]. The checklist contains eight items covering major domains of bias: population and research-context characteristics, specificity of clinical outcome definition, representativeness of the study sample, data-completeness reporting (whether numerator, denominator, and prevalence were all provided for each analytical stratum), and, most heavily weighted, case definition, namely, the method of pathogen detection and the level of detail in its description. This final criterion carries a maximum score of two; the overall possible score is 9. Records scoring 7.5 or greater were considered as good quality/low risk of bias; 5–7.5 as moderate quality/moderate risk; and those under 5 as poor quality/high risk. The complete checklist and scoring schema are presented in Supplementary Table S6. Final score was assigned based on the average from three reviewers. Public health surveillance datasets were not rated, and treated as having a moderate risk of bias. Records providing only information on genotypic distribution were not rated either.

### 2.4. Prevalence Data Analysis

Random-effects meta-analyses and meta-regression were performed on the log-odds scale using the restricted maximum-likelihood estimator with Hartung–Knapp–Sidik–Jonkman adjustment for confidence intervals, as implemented in the metafor package [25] in R v4.5.1. Pooled prevalences are given as percentages with corresponding 95% confidence intervals (95% CI) on the percentage scale. The $I^{2}$ and $\tau^{2}$ statistics were used as measures for relative and absolute between-study variance, respectively. Separate pooled estimates were obtained for all entries combined and for each major analytical stratum, and also for biennial bins. The 95% confidence intervals for single studies were calculated using the logit-transformation method.

Sensitivity analyses were conducted to assess the robustness of the pooled estimates: (a) restricting the analysis to records rated as high quality/low bias and without age-bridging categories; (b) excluding entries corresponding to non-dominant levels of major study factors (i.e., factor with alternate levels comprising at least one-fifth of all entries), testing for effect of each factor separately; and (c) restricting the analysis to high-quality studies and dominant major factor levels. Pooled estimates from the sensitivity models were compared with the main analysis to evaluate stability of results and changes in heterogeneity ($I^{2}$ and $\tau^{2}$) and prevalence estimates.

The effect of clinical presentation on RSV prevalence was assessed via meta-regression using clinical category (ARI, LRTI, or pneumonia) as a categorical moderator. The effect of study scale was similarly assessed, with scale approximated by the logarithm of study sample size (${log}_{10}\left(n\right)$). Two models were fitted: a main-effects model including ${log}_{10}\left(n\right)$ and temporal stratum as moderators, and a more complex model replacing global ${log}_{10}\left(n\right)$ slope with stratum-specific slopes. Model fit was compared using the Bayesian information criterion (BIC).

### 2.5. Genotypic Distribution Analysis

Data on RSV subtypes, genotypes, or clades were summarized as absolute counts and relative frequencies within each defined geographic region. Additional information on the distribution of circulating RSV variants in Russia was retrieved from the VGARus database [26] (Supplementary File S1), which aggregates pathogen and parasite genomic sequences originating in the Russian Federation. All RSV-related records with identified lineage from the latter source were extracted together with their associated metadata. When a study reported genotypes and had corresponding sequences available for retrieval (either as Supplementary Files or deposited in the GenBank or VGARus databases), we inferred clades using the Nextclade web service (v.3.18.1, commit: 15d409c) [27]. If sequences were not available, we assigned A.D clade to all sequences annotated as ON1, and B.D clade to BA-lineage genotypes.

## 3. Results

### 3.1. Retrieved Records Characteristics

After initial filtering, deduplication, and full-text screening, 68 eligible studies were retained, contributing the total of 360 eligible prevalences for further analysis (Figure 1, Supplementary Table S3). The most common reasons for exclusion at the title-and-abstract screening stage were a focus on bovine RSV or infection severity; at the full-text level, exclusions were mostly due to non-eligible article type (40 reviews and 1 case report) or absence of extractable RSV prevalence data (39 studies).

The earliest data collection dated to 1997 and corresponded to an IgM-based serologic investigation in Georgia [28]; the most recent extended to November 2025, and included nation-scale epidemiological surveillance datasets [20,21,22]. Together, the corpus of included studies spanned through, although disjointly, nearly three decades of coverage. Papers ranged from 2006 to 2025, with only 3.2% appearing before 2010 and roughly equal proportions published during the 2010s and 2020s (46.8% and 50.0%, respectively).

**Figure 2:**
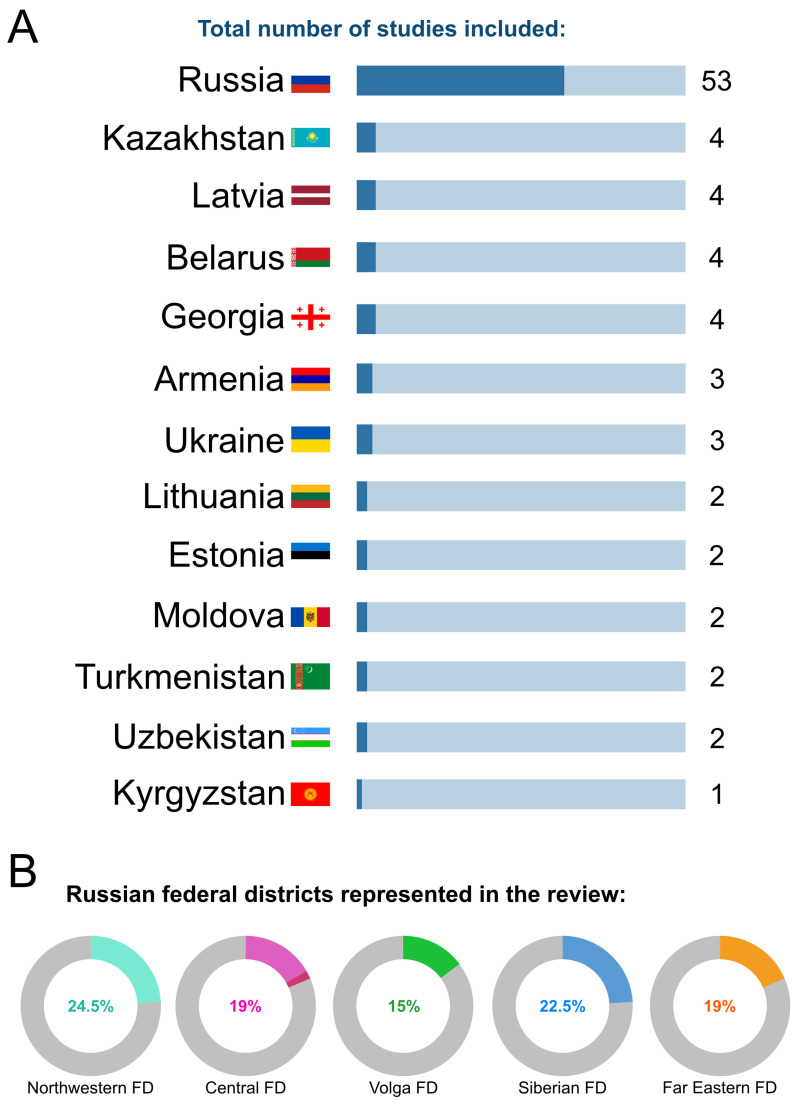
Regional distribution of studies included in the review. (**A**) Number of included studies by country of origin. (**B**) Donut-charts of Russian federal districts (FDs) represented in the review out of all the studies conducted in Russia. One study covering the “European region” was grouped into Central FD.

Records originated from 13 post-Soviet countries: fifty-three records reported data from the regions of Russia; six records covered the Baltic States; four each covered Kazakhstan, Belarus, and Georgia; three each Ukraine and Armenia; two each Moldova, Turkmenistan, and Uzbekistan; and one Kyrgyzstan (Figure 2A). Within Russia, coverage extended across nearly all federal districts, with only North Caucasian and Southern federal districts lacking dedicated RSV prevalence study. Research clustered mainly, and with comparable representation, in Northwestern, Siberian, Far Eastern, Central, and Volga federal districts, which together accounted for about four-fifths of all records, while the Ural federal district was represented by a single study focusing on RSV clade distribution (Figure 2B). Data from Central, Northwestern, and Siberian federal districts originated mainly from their major regional centers—Moscow, St. Petersburg, and Novosibirsk. In the Far Eastern federal district, most entries (6) were from Khabarovsk krai, with other locations (Sakha Republic, Primorsky and Zabaykalsky krais, and the Amur and Sakhalin oblasts) providing roughly even coverage across the area. The Volga federal district showed the most uniform sampling, with Tatarstan Republic, Nizhny Novgorod, and Saratov oblasts each contributing two entries, while Kirov, Penza, and Orenburg oblasts were represented by single entries (see Appendix A Table A1 and Supplementary Table S3). Sixteen studies in Russia were multicentered, encompassing multiple cities or regions; all four records from Kazakhstan were multicentered as well, as were one from Belarus and one from Georgia. Two records spanned multiple FSU countries: the FluNet dataset [22] and a study reporting The European Surveillance System outputs [29].

PCR was the dominant RSV detection technique, employed in 63 of 68 studies. Alternative methods included anti-RSV IgM serology (two studies) and immunofluorescence-based assays (five studies). Two studies combined multiple techniques, one of them using PCR alongside other methods. By the second half of the 2010s, all non-PCR techniques had disappeared from practice, with the final use of an alternative approach reported in 2019 in Armenia [30].

Overall, 39 studies examined children and 29 mixed-age groups, with 7 also including senior adults. Forty-nine studies investigated inpatients, one study examined outpatients exclusively, and 18 analyzed mixed in- and out-patient settings. Only a single study addressed asymptomatic community participants [31]. For most records, data could be consistently extracted only for cases classified as ARI. A small subset of 13 studies distinguished specific diagnosis—pneumonia, bronchiolitis, or any LRTI.

Most of the examined records reported only marginal prevalences of RSV, with no regard to co-infections. Those nine which had information on co-detections largely reported the number or percentage of mixed infections, with no data on the prevalence of specific pairings between RSV and other respiratory pathogens.

Risk of bias was assessed using a custom quality checklist (see Section 2 and Supplementary Tables S3 and S6 for actual scores), and the score, averaged through three raters, ranged from 4.83 to the maximum of 9. Thirty-seven studies were rated high quality, 23 moderate, and two studies rated as low quality. Inter-rater reliability for quality score assignment was good, with an intraclass correlation coefficient of 0.781 (95% CI 0.691–0.853, two-way random, absolute agreement model). Three public health surveillance datasets were not rated and were considered being of moderate risk of bias. Three studies that reported only RSV genotypic distribution were not rated either, because they were not considered for the prevalence analysis.

### 3.2. RSV Prevalence in Different Age Groups

**Figure 3:**
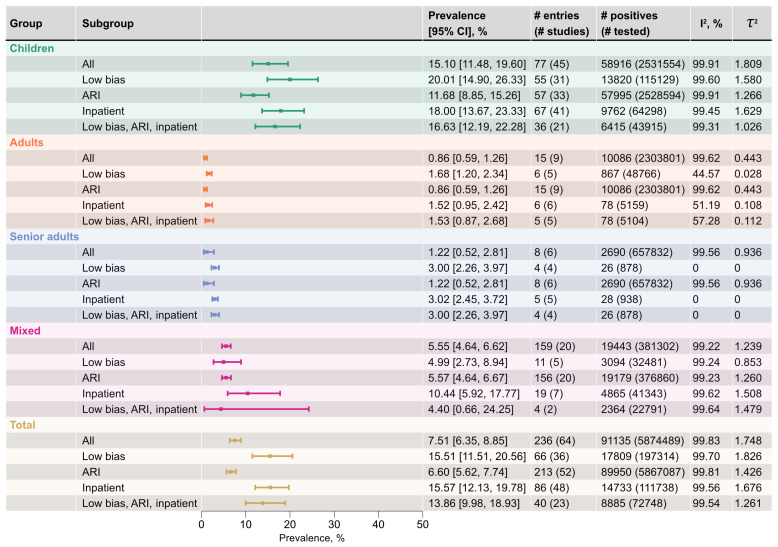
Pooled RSV prevalence across different age strata and sensitivity analyses. Prevalence estimates and 95% confidence intervals (95% CI) were derived from random-effects meta-analysis (REML estimator with Hartung–Knapp–Sidik–Jonkman adjustment). “All” denotes the overall pooled estimate within each age stratum. “Low bias” denotes estimates after excluding records rated moderate or low quality. “ARI” denotes estimates restricted to entries classified as ARI. “Inpatient” denotes estimates after excluding entries corresponding to outpatient or mixed in-/out-patient settings. “Low bias, ARI, inpatient” denotes estimates corresponding to ARI inpatients from low-bias studies.

**Figure 4:**
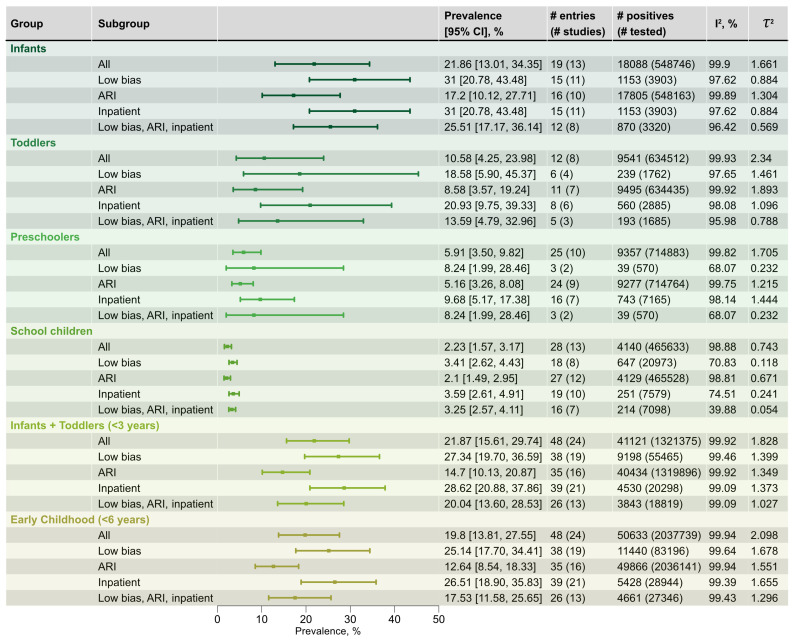
Pooled RSV prevalence across different age strata in children and sensitivity analyses. Prevalence estimates and 95% confidence intervals (95% CI) were derived from random-effects meta-analysis (REML estimator with Hartung–Knapp–Sidik–Jonkman adjustment). “All” denotes the overall pooled estimate within each age stratum. “Low bias” denotes estimates after excluding records rated moderate or low quality. “ARI” denotes estimates restricted to entries classified as ARI. “Inpatient” denotes estimates after excluding entries corresponding to outpatient or mixed in-/out-patient settings. “Low bias, ARI, inpatient” denotes estimates corresponding to ARI inpatients from low-bias studies.

Across all regions and age strata, 5,874,489 participants presenting with ARI were included in the analysis. Overall, 7.51% (95% CI 6.35–8.85%; 64 studies) tested positive for RSV (Figure 3). Among all age strata, the highest pooled prevalence was observed in the joint infant and toddler group (<3 years), which reached 21.87% (95% CI 15.61–29.74%; Figure 4), with pooled pediatric RSV prevalence averaging to 15.10% (95% CI 11.52–19.54%). The lowest prevalence was observed in adults (18–64 years) and senior adults (65+ years), with pooled estimates of 0.86% (95% CI 0.59–1.26%) and 1.22% (95% CI 0.52–2.81%), respectively. Studies conducted in mixed or unspecified age setting showed an intermediate prevalence of 5.55% (95% CI 4.64–6.62%), consistent with their predominantly pediatric composition as described by authors of those studies in most of the cases. As anticipated, RSV occurred most frequently in young children, particularly those under six years of age. A finer pediatric stratification highlighted a gradual decline in prevalence with age: from 21.86% (95% CI 13.01–34.35%) in infants to 2.23% (95% CI 1.57–3.17%) in school age children, approaching adult levels. Each intermediate category displayed progressively lower prevalence (Figure 4). The joint group of infants and toddlers, as has been mentioned before, ranked first, and when preschoolers were added (<6 years overall, early childhood), the pooled prevalence remained slightly decreased to 19.80% (95% CI 13.81–27.55%), confirming these youngest children as the most vulnerable to RSV infection.

Across all strata, heterogeneity was high ($I^{2}>98.00\%$), with the between-study variance ($\tau^{2}$) on the log-odds scale ranging from 1.809 and 1.748 in the joint pediatric and overall strata to 0.443 in adults under the broad age stratification. Within pediatric cases, $\tau^{2}$ varied from 2.340 in toddlers to 0.743 in school-age children, with infants having intermediate variance of 1.661. Excluding low-quality and bridging records (Figure 3 and Figure 4, “Low bias” rows) substantially reduced absolute heterogeneity but, in most strata, did not materially change relative heterogeneity. Only in adults and senior groups did $I^{2}$ drop markedly to 44.57% and 0.00%, respectively, which reflects the very small number of contributing entries (six and four). Removing high-risk-of-bias records also increased pooled prevalence estimates across most strata; however, the confidence intervals overlapped substantially, suggesting that this apparent increase was not statistically significant. Pediatric sub-strata mirrored the described pattern (Figure 3 and Figure 4, “Low bias”).

Studies rated as moderate or low quality encompassed 5,677,175 participants, which comprised 96.64% of all individuals tested for RSV diagnosed with ARI, and, thus, had a disproportionate impact on the overall pooled estimates. Of particular interest are three nation-scale surveillance records [20,32,33]. These large datasets, which utilized mixed in-/out-patient setting, report significantly lower RSV prevalence compared to more localized studies: 0.92% (n=4,667,302, 95% CI 0.91–0.93%) [32], 6.49% (n=117,987, 95% CI 6.35–6.63%) [33], and 1.79% (n=642,988, 95% CI 1.75–1.82%) [20] overall RSV prevalence. The influence of these records suggests that differences in study scale—local cohorts vs. global surveillance outputs—represent a primary source of heterogeneity in reported RSV prevalence (see Section 3.4. for details).

Excluding records that reported clinical presentation other than ARI (Figure 3 and Figure 4, “ARI” rows) led to a decrease in absolute heterogeneity and only marginal decreases in relative heterogeneity. For most strata, restricting analyses to ARI cases produced lower pooled prevalences, although the differences were not statistically significant. Restricting the dataset to inpatient setting (Figure 3 and Figure 4, “Inpatient” rows) also reduced heterogeneity, but to a lesser extent than the preceding exclusions. Finally, narrowing the data to low-bias ARI inpatients yielded the lowest absolute heterogeneity in nearly all strata, while relative heterogeneity decreased only slightly (Figure 3 and Figure 4, “Low bias, ARI, inpatient”). The mixed-age group was an exception: decline occurred only after low-quality entries were removed; other manipulations increased absolute between-study variance.

### 3.3. RSV Prevalence in Different Clinical Presentations

Clinical presentation demonstrated the most consistent relationship with pooled prevalence estimates and was, therefore, examined as a potential moderator of RSV prevalence in subsequent analyses. Accurate moderation analysis requires adequate representation of each outcome; therefore, we limited analysis to strata having at least five entries for any outcome other than ARI. This criterion was met only for the infants and toddlers and joint early childhood groups (the latter extended by preschoolers), and also for the aggregated pediatric stratum. As the infants and toddlers and early childhood groups showed similar prevalence estimates (Figure 4), only the early childhood group was analyzed.

**Figure 5:**
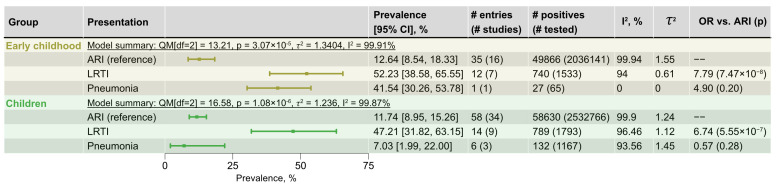
Pooled RSV prevalence by clinical presentation within pediatric strata. Prevalence estimates and 95% confidence intervals (95% CI) were derived from random-effects meta-regression using clinical presentation as moderator with ARI as a baseline (REML estimator with Hartung–Knapp–Sidik–Jonkman adjustment). For each age stratum, summary statistics for the full model using clinical presentation as a moderator are reported ($\mathrm{QM}\left[\mathrm{df}=2\right]$, $p\left(\mathrm{QM}\right)$, $\tau^{2}$, $I^{2}$). Clinical presentation categories include “ARI”, “LRTI”, and “Pneumonia”. “Early childhood” denotes children under 6 years of age. “Children” denotes all individuals under the age of 18.

Within the early childhood group, pooled RSV prevalence varied by clinical presentation, ranging from 12.64% (95% CI 8.54–18.33%) for ARI to 52.23% (95% CI 38.58–65.55) in combined LRTI diagnoses. Meta-regression confirmed a significant overall moderation by clinical presentation ($\mathrm{QM}\;[\mathrm{df}=2]=13.21,\;p\left(\mathrm{QM}\right)=3.07\times 10^{-5}$), with prevalence increasing in cases of LRTI and pneumonia (Figure 5). Residual heterogeneity remained ($I^{2}=99.91\%$ for ARI, and 94.00% for LRTI), and $\tau^{2}$ decreased by 0.94 log-odds units toward LRTI, indicating that factors beyond case definition contribute to variability. Pneumonia was represented by a single entry, preventing reliable characterization of its effect in meta-regression. The only significant pairwise contrast was ARI vs. LRTI. A similar, but slightly attenuated, pattern was observed for the broader pediatric stratum, with one exception: contrary to expectations, entries reporting prevalence among pneumonia cases demonstrated a lower pooled prevalence (7.03%, 95% CI 1.99–22.00%) than in ARI (11.74%, 95% CI 8.95–15.26%).

### 3.4. RSV Prevalence over Time

**Figure 6:**
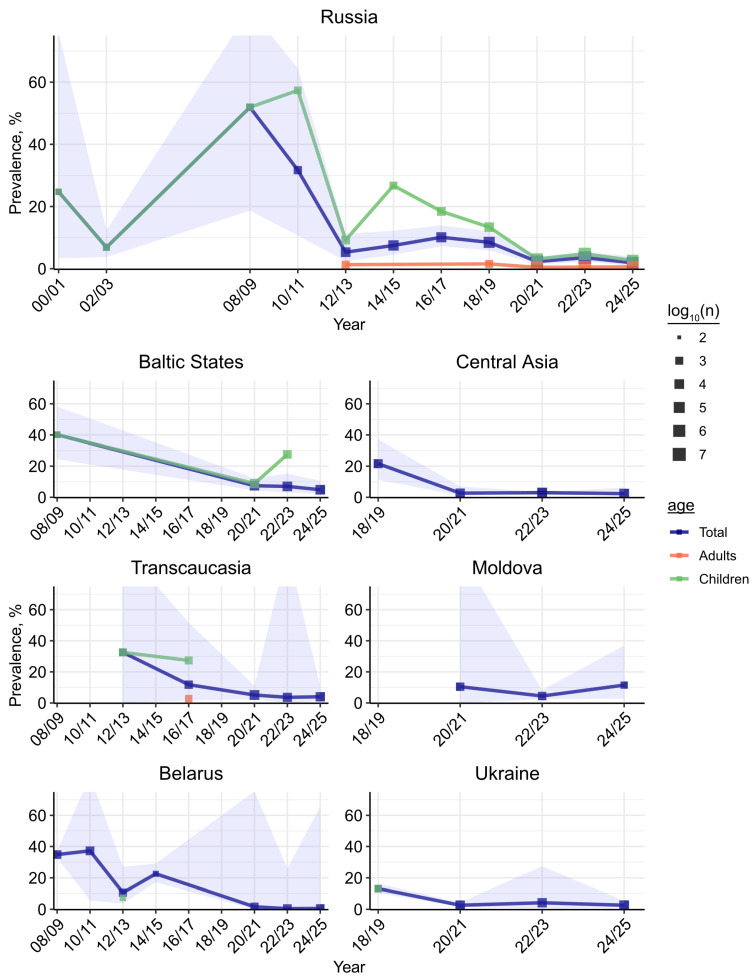
Temporal distribution of RSV prevalence across different regions of FSU. RSV prevalences were pooled across two-year bins separately for three different age groups by random-effects meta-analysis (REML estimator with Hartung–Knapp–Sidik–Jonkman for confidence intervals). Pooling was based on the floored midpoint year of entry observation interval, with disjoint (by at least one year) intervals, and those spanning more than 3 years omitted from the analysis. Points denote prevalences; their sizes reflect the logarithm of pooled sample size. The blue shading denotes the 95% confidence interval for the overall prevalence estimate (“Total”). “Children” denotes estimates for individuals under 18 years; “Adults” denotes estimates for individuals of age 18 or older.

**Figure 7:**
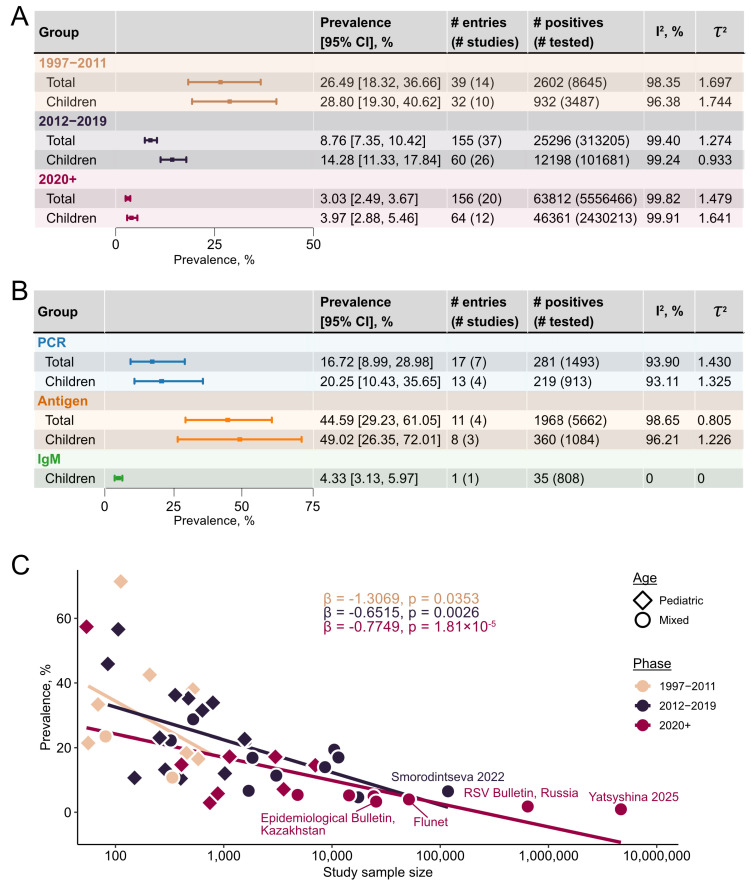
Temporal features of RSV prevalence in the FSU. (**A**) Pooled RSV prevalence estimates across distinct temporal strata in children and total population. (**B**) Effect of detection method on pooled RSV prevalence in joint pediatric and overall strata during the 1997–2011 period. (**C**) Effect of study scale on pooled RSV prevalence across distinct temporal strata. X-axis denotes study sample sizes; Y-axis denotes prevalence. Straight lines give linear phase-wise trends. Regression coefficients (β) are derived from the meta-regression model fitted as described above, including the phase and the interaction between the phase and the logarithm of the study sample size. Study-specific slopes are given in log-odds scale.

RSV prevalence varied substantially across the timespan covered by the review, with Russia providing the most informative temporal signal owing to its extensive representation in the dataset. Three phases can be distinguished based on differences in prevalence patterns (Figure 6 and Figure 7A): the earliest (1997–2011), characterized by the highest reported RSV prevalence and uneven sampling; the intermediate phase (2012–2019), marked by diagnostic standardization toward PCR, more consistent temporal sampling, and moderate RSV prevalence; and the closing phase (2020 onward), defined by the COVID-19-related global minimum of RSV prevalence, yet enriched by newly available nation-scale surveillance data. The earliest phase demonstrated the global maximum of RSV prevalence across all geographical strata: 51.19% (95% CI 18.68–83.54%) in the 2008/2009 biennium in Russia. This biennial bin also encompassed the global maximum for the Baltic States (40.17%, 95% CI 24.45–58.22%) and the second-highest biennial prevalence in Belarus (34.83%, 95% CI 32.74–36.99%). Key features of this phase were the predominance of pediatric cases and substantial heterogeneity in detection techniques. Of the 14 studies from this phase, nine used PCR and five employed alternative or combined techniques (antigen detection assays or IgM testing); one [34] confirmed RSV infection through a combination of PCR, immunochromatography, and serological testing. In the 2010s, this methodological diversity had disappeared, with the latest non-PCR technique reported in 2019 in a study from Armenia spanning the 2013/2014 epidemiological season [30]. Methodological differences proved a major driver of early phase variability. Pooled RSV prevalence among PCR-based studies was 20.25% (95% CI 10.43–35.65%) for pediatric cohorts and 16.72% (95% CI 8.99–28.98%) overall, while antigen assays yielded 49.02% (95% CI 26.35–72.01%) and 44.59% (95% CI 29.23–61.05%), respectively. The single IgM-based study reported a prevalence of 4.33% (95% CI 3.13–5.97%) (Figure 7B). The only study employing a composite case definition (RSV confirmed by a positive signal from any of PCR, IgM serology, or antigen detection test) [34] yielded a pooled prevalence of 68.78% (95% CI 43.33–86.39%) in pediatric LRTIs. The unexpectedly higher detection rates observed with antigen-targeted assays are more plausibly attributed to confounding from pediatric LRTI cohorts than to intrinsic assay sensitivity. Overall, the elevated prevalence observed during this phase likely reflects an enrichment in pediatric LRTI cases: this phase alone accounted for approximately 40% of all pediatric LRTI cases across the entire dataset.

The intermediate phase (2012–2019) presented a markedly different epidemiological landscape, with a pooled phase-wise prevalence of 14.28% (95% CI 11.33–17.84%) for pediatric strata and 8.76% (95% CI 7.35–10.42%) overall (Figure 7A). The first biennium encompassed a local minimum for Russia and Belarus; thereafter, overall RSV prevalence in Russia stabilized, fluctuating between 5% and 12%. Pediatric strata had seen a sharp rise in the subsequent biennium following the 2012/2013 local minimum (26.73%, 95% CI 14.96–43.08%, *n* = 962), with prevalence then gradually declining toward the global RSV prevalence minimum that tags the start of the closing phase (Figure 6). The intermediate phase was also the first to be captured by nation-scale surveillance systems, although the relevant outputs were published retrospectively, after the phase had concluded [33,35,36]. Despite constituting only a small fraction of studies, these nation-scale datasets account for roughly two-thirds of all tests performed in this phase and may, therefore, have a disproportionate impact on the pooled estimates.

The 2020/2021 biennium, marked by the rise of the COVID-19 pandemic, saw the global minimum for RSV prevalence: 2.48% (95% CI 1.18–4.48%) overall and 3.30% (95% CI 1.30–8.11%) among children in Russia, with comparable estimates in other regions. Subsequent biennial bins showed a modest increase to 2.98% overall and 4.06% in children, with similar temporal patterns observed across finer-scale geographical strata, although with diminished coverage (Figure 6 and Figure S1). This closing phase, which remains ongoing, is characterized by the richest temporal and geographical coverage to date, including data for most of the FSU countries, and covers 94.52% of all performed tests. The wide availability of nation-scale surveillance outputs is the principal source of this coverage, offering weekly resolution on RSV prevalence in a subset of the FSU countries [20,21,22]. These surveillance outputs comprise 98.89% (*n* = 5,494,971) of tests in the ongoing phase, rendering pooled prevalence virtually synonymous with surveillance-derived estimates (Figure 7A).

The three phases, thus, differ not only in prevalence but also in the nature of the contributing studies. Chiefly, the number of participants tested for RSV increased considerably: from 8645 in the earliest phase, the longest in duration, to 5.5 million in the closing phase, which spans only six years. This shift reflects a transition from local small-scale studies to nation-scale surveillances, rendering study scale an evident source of heterogeneity. To test this, we conducted a meta-regression with phase and the logarithm of the study sample size (${log}_{10}\left(n\right)$) as moderators. The omnibus test was significant ($\mathrm{QM}\left[\mathrm{df}=3\right]=23.2703,\;p\left(\mathrm{QM}\right)=3.55\times 10^{-9}$); phase itself was not a significant moderator, ${log}_{10}\left(n\right)$ was strongly associated with prevalence ($\beta =-0.7519,\;p=1.92\times 10^{-7}$), indicating that larger studies tend to report lower RSV prevalence. A subsequent model allowing the ${log}_{10}\left(n\right)$ slope to vary between distinct phases did not improve fit relative to the original model (ΔBIC=3.5824). Nonetheless, phase-specific slopes were negative and significant: earliest (β=−1.3069, p=0.0353), intermediate (β=−0.6515, p=0.0026), and closing ($\beta =-0.7749,\;p=1.81\times 10^{-5}$; Figure 7C). The steeper slope observed in the earliest phase is consistent with the predominance of small, pediatric-focused cohorts capturing high-prevalence populations, while the attenuated slopes in later phases likely reflect the homogenizing influence of large-scale surveillance data, although the difference between slopes should be interpreted cautiously given the absence of improved model fit. Restricting analysis to pediatric studies alone yielded a significant main-effects model ($\mathrm{QM}\left[\mathrm{df}=3\right]=3.7524,\;p\left(\mathrm{QM}\right)=0.0243;\;\beta_{{log}_{10}\left(n\right)}=-0.9272,\;p=0.0201$); however, the more complex model with phase-specific slopes lacked sufficient power to reject the null hypothesis, although all slopes remained negative.

### 3.5. RSV Prevalence Monthly

**Figure 8:**
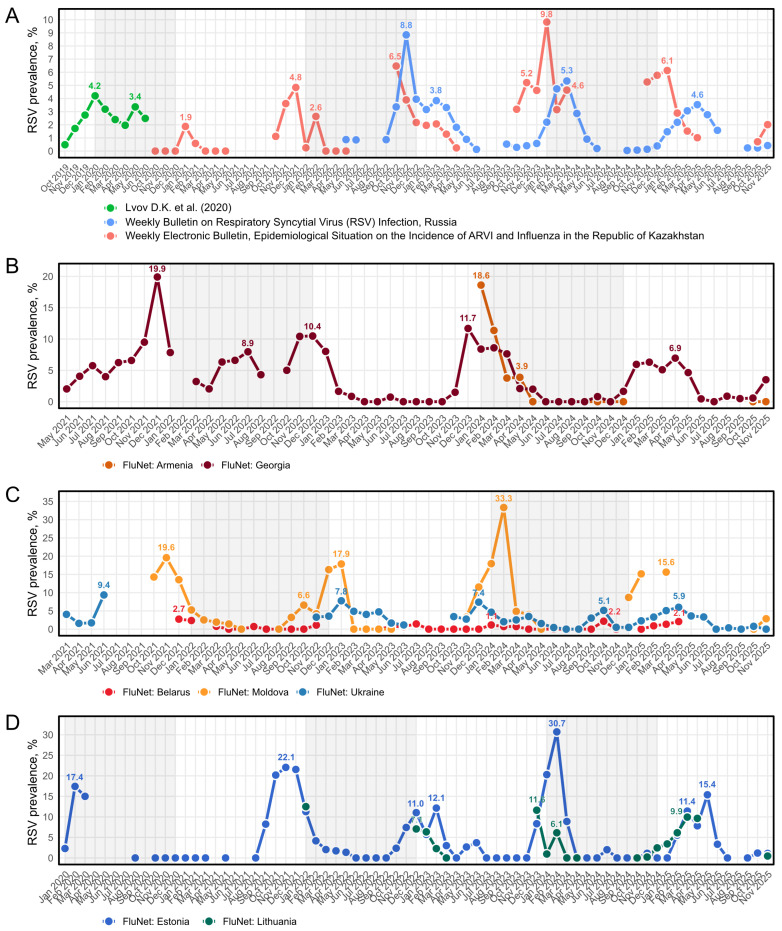
Monthly RSV prevalence in FSU countries. (**A**) Monthly RSV prevalence in Russia and Kazakhstan from October 2019 to November 2025. Monthly data on RSV-prevalence was acquired from Lvov et al. (2020) [37], depicted in green; The Weekly Bulletin on RSV infection from Smorodintsev Research Institute of Influenza [20], depicted in blue; and The Weekly Electronic Bulletin, Epidemiological Situation on the Incidence of ARVI and Influenza in the Republic of Kazakhstan [21], depicted in red. Gray boxes highlight every other year (January–December). Numbers denote prevalences for the observed local maxima. (**B**–**D**) Monthly RSV prevalence acquired from the WHO FluNet platform [22]. X-axis indicates month and year (Mon 20XX); Y-axis indicates RSV prevalence (%). Gray boxes highlight every other year (January–December). Numbers denote prevalences for the observed local maxima.

Most of the studies did not report seasonal patterns of RSV distribution. Among those that considered RSV seasonality, only three studies included RSV prevalence with monthly resolution, necessary to adequately trace its seasonal dynamics; the rest were suitable only for broader, comparative analyses. Three sources, one cross-regional research (2019–2020, the time series by Lvov et al.) [37], the weekly bulletin on RSV prevalence from Russia (2022–2025) [20], and the weekly ARI surveillance bulletin from Kazakhstan (2020–2025) [21], provided detailed monthly data, overall covering the period from October 2019 to November 2025, allowing for characterization of seasonality patterns (Figure 8A). The extracted data, including total number of studied samples and PCR-detected RSV-positive cases, can be seen in Supplementary Table S7. The time series by Lvov et al. [37], representing the last pre-COVID-19 season, can be seen as a baseline for RSV circulation dynamics. It showed a primary peak in January 2020 (4.2%, 95% CI 3.38–5.23%), before the first COVID-19 case was reported in Russia, and a secondary rise in May 2020 (3.4%, 95% CI 2.32–4.86%). The next monthly observations for RSV prevalence followed only in the 2022/2023 season, which encompassed the COVID-19 pandemic decline and the official WHO declaration of the end of the COVID-19 global health emergency in May 2023 [38]. This season had an earlier RSV maximum in November (8.85%, 95% CI 7.96–9.82%), and a minor late-winter peak in February–March (3.8%, 95% CI 3.5–4.2%). A similar two-peak structure appeared in Kazakhstan during the same season, but with the initial maximum occurring a month earlier in October (6.47%, 95% CI 4.82–8.64%). Kazakhstan retained this early-season peak in 2023/2024 (5.2%, 95% CI 4.23–6.4%), although it was almost twice smaller than its major peak in January 2024—the highest recorded prevalence in the seasonality dataset (9.82%, 95% CI 7.4–12.94%), while according to data from Russia, only the February–March maximum was observed (5.34%, 95% CI 5.04–5.65%). By 2024, RSV dynamics appeared to have returned to its pre-pandemic baseline. In 2025, Kazakhstan displayed its characteristic January peak (6.13%, 95% CI 5.03–7.46%), while Russia had shown a delayed rise in April (3.53%, 95% CI 3.35–3.73%). At the time of writing, public-health surveillance systems in both countries report the initial winter rise typical of RSV.

To assess regional patterns of RSV seasonality, we expanded our analysis using sentinel surveillance data from the WHO FluNet platform [22]. To ensure sufficient resolution, we included only data points where at least 25 individuals were tested per month. Data meeting these criteria were available for seven countries in the FSU region: Armenia, Georgia (Figure 8B), Belarus, Moldova, Ukraine (Figure 8C), Estonia, and Lithuania (Figure 8D). Countries with insufficient testing volumes and sparce temporal coverage were excluded from the primary analysis (Figure S2).

A common feature was an unusually intense and early RSV peak in the autumn–winter of 2021, followed by a gradual return to more traditional winter circulation patterns in subsequent years. Georgia, Moldova, and Estonia all experienced a major RSV wave in late 2021, with pronounced peaks occurring between November and December (Georgia: December 2021, 19.9% (95% CI 15.06–25.84%); Moldova: November 2021, 19.6% (95% CI 12.86–28.68%); Estonia: November 2021, 22.1% (95% CI 15.87–29.80%)). This was followed by more varied activity, notably, an uncharacteristic smaller peak in Georgia in July 2022 (7.9%, 95% CI 4.87–12.81%) and a bimodal (two-peak) structure in Estonia during the 2022–2023 winter (December 2022: 11.0% (95% CI 7.87–15.26%); February 2023: 12.1% (95% CI 7.19–19.80%)). By the 2023–2024 season, all three countries demonstrated a return to a strong winter peak, primarily in December in Georgia (11.7%, 95% CI 7.28–18.22%) and in February in Moldova and Estonia (Moldova: 33.3% (95% CI 18.34–52.67%); Estonia: 30.7% (95% CI 23.31–39.25%)). Lithuania showed consistent winter peaks, primarily in December 2022 (7.1%, 95% CI 3.20–14.83%), December 2023 (11.5%, 95% CI 4.92–25.05%), and February 2024 (6.1%, 95% CI 4.39–8.50%). Armenia’s available data were characterized by a single, major peak in January 2024 (18.6%, 95% CI 14.92–22.97%).

In stark contrast, RSV prevalence in Ukraine and Belarus remained substantially lower throughout the observation period (Figure 8C). These countries exhibited only moderate, sporadic peaks, with Belarus rarely exceeding 1% monthly prevalence. The most notable activity in this group was observed in 2023 in Ukraine with a peak in January (7.8%, 95% CI 6.07–9.90%) and December (7.4%, 95% CI 5.47–9.92%). Notably, an early peak was observed in October 2024 in both Ukraine (5.1%, 95% CI 3.26–8.01%) and Belarus (2.2%, 95% CI 0.30–13.88%), a pattern that was uncharacteristic of other countries in the region.

A notable shift in seasonality was observed across all analyzed countries in 2025, with the typical winter peak transitioning into early spring, specifically, a March peak appearing in Moldova (15.6%, 95% CI 6.66–32.47), Estonia (11.4%, 95% CI 7.83–16.35%), and Lithuania (9.9%, 95% CI 8.29–11.89%). An April peak was observed in Georgia (6.9% (95%, CI 3.90–12.13%), Ukraine (5.9%, 95% CI 4.17–8.55%), and Belarus (2.1%, 95% CI 0.29–13.36%). Estonia exhibited a subsequent late-season peak in May (15.4%, 95% CI 8.95–25.17%).

### 3.6. RSV Clades Distribution

Eight studies reported data on RSV subtype and clade distribution (see Supplementary Table S4). The genotyping dataset encompassed Latvia and four locations in Russia: Moscow and St. Petersburg (Central and Northwestern federal districts), and Ekaterinburg and Novosibirsk (Ural and Siberian federal districts). The dataset was further expanded by the RSV sequences deposited in the VGARus database, adding two more locations—Orenburg (Volga federal district) and the Republic of Kabardino-Balkaria (North Caucasian federal district), each contributing a single sequence. In total, the merged dataset comprised 297 unique RSV variants, with substantial overlap between the Moscow and Sverdlovsk samples from published studies and VGARus. Overall, the compiled dataset spans 2009–2024.

**Figure 9:**
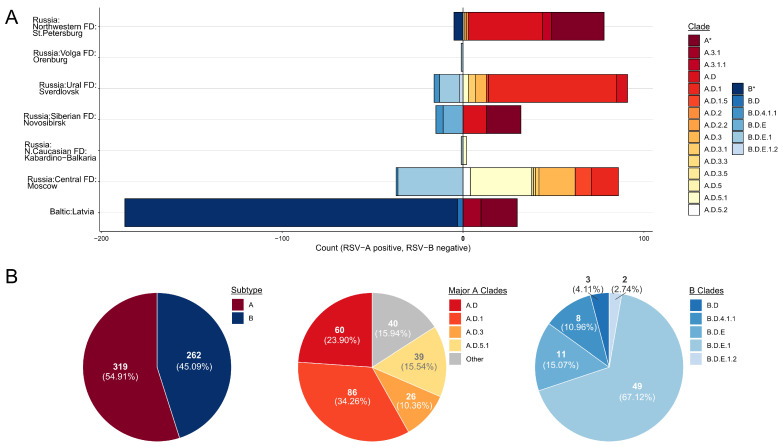
(**A**) Distribution of RSV subtypes and lineages across different sampling sites, based on data acquired from 8 studies and sequences deposited in the VGARus database. Unspecified clades of subtype A and subtype B are presented as A* and B*, respectively. (**B**) Summary pie charts depicting overall proportion of RSV subtypes, relative proportions of major RSV-A clades of RSV (in reds) and RSV-B clades (in blues).

Across this interval, RSV subtype A was detected most frequently; however, in the most recent study from Latvia [39], more than 90% of reported sequences were classified as subtype B (Figure 9B). Because that study did not generate new sequences, it provided no further information on the genotypic composition within the RSV-B, and RSV-A remained dominant across the observation window nearly in all other sources. Since the mid-2010s, lineage A.D (traditionally known as the ON1 genotype) has been recognized as the globally dominant cosmopolitan genotype. While A.3.1 and A.3.1.1 were detected in our dataset in 2009 and in 2013–2017, respectively, only A.D and its descendant clades were observed in subsequent years. Among these, lineage A.D.1 was the most frequently identified, largely due to extensive sampling in the Sverdlovsk oblast, but outside the Ural, no consistent pattern of dominance by any single clade was observed (Figure 9A and Figure S3).

For subtype B, only the cosmopolitan lineage B.D (traditionally known as the BA genotype) and its descendant clades were detected. In contrast to subtype A, subtype B displayed a clear dominance of a single lineage (B.D.E.1) during the past three years (Figure 9A and Figure S3).

## 4. Discussion

The review presents the first comprehensive synthesis of epidemiological data on RSV prevalence across the countries of the FSU, bringing together almost thirty years of research encompassing 65 studies and three nation-scale sentinel surveillance datasets from 13 countries. The dataset is dominated by records from different regions of Russia, but collectively outlines RSV epidemiology patterns for a population of nearly 300 million people, spanning diverse socioeconomics, climatic, and demographic contexts. Until now, this vast geographic area has been largely neglected in global synthesis of RSV epidemiology, with only a few systematic reviews including a very limited subset of studies, if any, often treating the region as a part of WHO’s Europe region [3,15,16,17]. As a result, data from FSU countries were either underrepresented or insufficiently evaluated; for example, unpublished data from Russia were classified as low quality in one review [3], and others contained too few records from the region to support robust analysis [15,16,17]. Moreover, these reviews lacked data from Central Asia and included little to no information from Transcaucasia, further limiting their ability to characterize RSV epidemiology across the FSU. By integrating previously fragmented data, this review establishes the first region-wide baseline for RSV epidemiology, highlighting both common patterns and geographic gaps, and providing a solid foundation for future surveillance and comparative work.

Most studies in the compiled dataset focused on children, mirroring global trends in RSV research, where pediatric cohorts are traditionally studied more extensively because they represent the primary risk group [4,11,40]. The data demonstrate a clear age-related gradient in RSV prevalence, with younger children—infants, toddlers, and preschoolers—having the greatest RSV prevalence, reaching 21.86% in infants before twelve months of age, and 23.02% in the joint infant and toddler group (Figure 4). These observations are consistent with findings in pediatric cohorts from a wide range of regions [3,16,41,42]. Prevalence declined progressively with age, while overall pediatric prevalence was 15.10%; in older groups, it ranged between 0.52 and 2.81%. Among pediatric cases, RSV prevalence varied systematically with clinical presentation, reaching its maximum 52.23% in children under 6 years and 47.21% across childhood LRTIs. This outcome is anticipated: decades of accumulated research have established RSV as the chief cause of pediatric bronchiolitis [2,16,43].

Only six studies reported RSV prevalence for senior adults (65+ years), mostly alongside data on other age strata, leaving no dedicated research focused on seniors identified in the FSU. This scarcity reflects the global trends. Despite the growing recognition of senior adults as another high-risk group, and the fact that three RSV vaccines targeting this population have recently been market-approved, evidence on infection burden and clinical outcomes in this age stratum remains scarce (reviewed in [11]). In our dataset, RSV prevalence in older adults did not differ significantly from that in younger adults, which may reflect lower rates of hospital admissions for ARIs among the senior population. This peculiarity has also been reported in the meta-analysis covering RSV burden in developing countries, which found lower RSV-associated rates, but higher in-hospital case-fatality rates, in older adults [44]. Senior adult cohorts, thus, represent a potent vector for future RSV research, particularly within the FSU, where current evidence is severely lacking.

Temporal variation in RSV prevalence adds another dimension to the epidemiological profile across Russia and the FSU, reflecting both virological dynamics and evolving public-health systems infrastructure. From its highest point during the 2008/2009 seasons (51.19%), pooled RSV prevalence gradually declined to 8.05% over the following decade and to 1.90% at the time of writing. This prolonged decrease in the first decade after the global maximum likely reflects the increasing number and scale of studies rather than a true reduction in RSV circulation, as pooled sample size grew substantially through the inclusion of large multicenter surveillance and mixed in-/out-patient datasets. We specifically note that the decline in overall RSV prevalence parallels that observed in the pediatric stratum, suggesting that temporal trends are most likely shaped primarily by changes in pediatric data composition rather than by any broad epidemiologic suppression of the virus. The same period coincided with the introduction of palivizumab into clinical practice in Russia in 2010 (reviewed in [45]). However, because palivizumab is administered mostly to high-risk infants and primarily reduces severe disease and ICU admissions [46], its targeted use is unlikely to have substantially shifted population-level RSV prevalence.

To account for these temporal changes in study design, diagnostic practices, and surveillance intensity, we stratified our analysis into three phases. The boundaries of these phases, while necessarily somewhat arbitrary, are data-driven—selected based on observed temporal patterns in Russia, the most extensively covered country in the dataset, and we believe they approximate actual trends in RSV prevalence research across the FSU reasonably well. The earliest phase (1997–2011) was characterized by sparse temporal and geographical coverage, pronounced methodological diversity—including serology, antigen detection, and PCR assays—and a strong focus on pediatric populations. This combination likely contributed to both inflated pooled prevalence estimates and high between-study heterogeneity. Our analyses confirmed that differences in diagnostic methodology were associated with variability in pooled prevalence, underscoring the impact of diagnostic approaches on epidemiological estimates. Surprisingly, the highest pooled prevalence was observed among antigen-targeted assays—49.02% in the pediatric stratum and 44.59% overall—while PCR, generally regarded as the gold standard owing to its superior sensitivity and specificity [47,48], yielded 20.25% and 16.72%, respectively. This unexpectedly high detection rate for antigen-based assays is likely a consequence of confounding: pediatric LRTI cohorts—predominantly infants and toddlers, the age group most susceptible to RSV—provided most of the entries for this analysis.

The intermediate phase (2012–2019) marked a transition toward methodological standardization, with PCR becoming the predominant diagnostic tool, and a parallel increase in study sample size. These changes improved cross-study comparability and resulted in more stable prevalence estimates with reduced absolute heterogeneity. Notably, this phase was the first to be captured by the nation-scale surveillance systems, although the relevant outputs were published only after the phase had concluded. The closing phase (2020 onward) has been dominated by large-scale surveillance datasets spanning nearly all FSU countries. Most countries now report RSV data to the WHO via the FluNet platform; for Kazakhstan and Russia, however, we relied on outputs from their independent national sentinel surveillance systems, which largely overlap with their FluNet submissions but offer more detailed data. This phase offers the greatest volume of tests and the most granular (weekly) temporal resolution. The growing availability of such publicly accessible surveillance data represents an important milestone in regional public health infrastructure, enabling timely, policy-relevant monitoring of RSV circulation and informing decisions on prophylaxis deployment, hospital preparedness, and outbreak response.

The closing phase also coincided with the COVID-19 pandemic. The 2020/2021 period, marked by the emergence and global spread of SARS-CoV-2, had seen the decline in incidence of non-COVID respiratory viruses, including RSV, as supported by our analysis. The 2022/2023 and 2024/2025 biennia showed a moderate increase in RSV prevalence of up to 2.98% overall and 4.06% pediatric prevalence; however, the intraseasonal pattern during these years displayed striking variation, departing from the regular winter peaks observed before the pandemic. Established global epidemiological patterns suggest winter seasonality of RSV within the temperate Northern Hemisphere, demonstrating a dominant transmission mode in February [49,50]. Our analysis incorporated one cross-regional research study alongside nation-scale sentinel surveillance outputs: the weekly RSV surveillance bulletin from Russia [20] and the weekly ARI surveillance bulletin from Kazakhstan [21]. Despite data discontinuities in the selected inter-epidemic seasons (mostly months from July to September), this allowed us to reconstruct local RSV prevalence trends in Russia and Kazakhstan for a research season encompassing the period from October 2019 onward. Interestingly, we observed an early peak in RSV prevalence in October 2022 in Kazakhstan, followed by slow decline with a second smaller peak in March. A similar October peak was reported in a study conducted in Novosibirsk, a major city in southern Siberia, adjacent to Kazakhstan [51], suggesting a shared autumn onset across neighboring regions. While data from Russian national RSV monitoring suggest an early November peak in 2022, this trend gradually shifts to indicate autumn onset with peak RSV prevalence in February–March followed by a marked decline in May–June to approximately half the peak volume starting from 2023. The Smorodintsev Research Institute of Influenza maintains a weekly influenza bulletin that includes PCR-based RSV detection data, beginning from the 2012–2013 epidemic [20]. Although these historical data could not be extracted because they are presented only in graphical form, it indicated similar pre-pandemic patterns of RSV seasonality. Notably, we observed a transient elevation in RSV prevalence in the immediate post-COVID-19 period in both Russia and Kazakhstan, followed by a gradual regression toward 2019 baseline levels. We expanded our scope by incorporating WHO FluNet platform sentinel surveillance data for seven additional FSU countries: Armenia, Georgia, Moldova, Belarus, Ukraine, Estonia, and Lithuania. Despite lower data resolution compared to weekly bulletins from Russia and Kazakhstan, several distinct trends were observed spanning the 2020–2025 period. Our findings indicate inter-annual variability in peak timing and intensity across the FSU region. However, by 2025 there appears to be a trend toward seasonal normalization; specifically, the disappearance of a more typical December–February peak and a shift toward a later, milder spring peak, which align with the post-COVID seasonal patterns observed in Russia. The widespread implementation of non-pharmaceutical interventions to control COVID-19, including mask use, physical distancing, and mobility restrictions, substantially reduced opportunities for RSV transmission, resulting in a marked suppression of circulation. Following the relaxation of these measures, RSV activity progressively reverted to its characteristic pre-pandemic seasonal pattern. Similar dynamics, to varying degrees, have been reported for other respiratory viruses and are described in a recent meta-analysis encompassing 31 countries, supporting the notion that non-COVID-19 respiratory viruses are re-establishing their pre-pandemic seasonality after a temporary post-pandemic surge [52]. Importantly, this analysis showed that post-pandemic viral resurgence was not synchronous but followed a structured sequence, with rhinoviruses re-emerging first, followed by RSV, seasonal coronaviruses, adenoviruses, and parainfluenza viruses, whereas influenza A/B and metapneumovirus recovered later.

Data on distribution of RSV antigenic subtypes and genotypes were limited to two countries: Russia and Latvia, with the latter reporting mostly subtypes information. In Russia, RSV-A appears to be more frequently detected, while Latvia’s variants were predominantly RSV-B. The proportion of RSV-A and RSV-B vary substantially across regions and study periods and, overall, remains approximately balanced [2,53,54]. Such variability complicates the prediction of subtype behavior and emphasizes the need for continuous, multiseason surveillance. The dominant genotypes for both subtypes correspond to cosmopolitan lineages, distinguished by the well-characterized duplications in the G protein ectodomain—A.D and B.D (ON1 and BA under traditional classification) [54,55]. These lineages displaced almost completely all other circulating genotypes by the second half of 2010s. Genotypes circulating before the emergence of B.D were not detected within the FSU; however, non-duplication A.3.1 and A.3.1.1 persisted [2,54]. From 2017, all identified RSV A variants in Russia belonged to the A.D lineage or its descendant clades, with no consistent genotype dominance for any of them. By contrast, within RSV-B, the B.D.E.1 clade was the most common, consistent with global RSV-B lineages distribution, showing its predominance since 2022 [56,57,58]. Taken together, these findings underscore several important epidemiological and surveillance trends in the region. Although our study benefits from access to the VGARus national system, which includes a considerable number of sequences absent from GenBank and GISAID, RSV genomic data from the FSU territories remain limited in both quantity and geographic coverage. Compared with the overall dataset available for the WHO European Region, the volume of Russian RSV sequences remains disproportionately low. This limitation reflects a broader structural gap in genomic surveillance that, if unaddressed, may delay the detection of emergent variants and limit comparative analyses between countries. Improving sequence deposition practices and expanding sequencing capacity should, therefore, be considered essential priorities for future public health efforts.

Limited geographical coverage extends beyond the genotypic distribution analysis. The compiled dataset includes no studies from Azerbaijan and Tajikistan, and the remaining post-Soviet countries included in the review (Ukraine, Belarus, Moldova, Estonia, Latvia, Lithuania, Armenia, Georgia, Kazakhstan, Uzbekistan, Turkmenistan, and Kyrgyzstan) contribute only a small fraction of entries, compared to Russia, and predominantly in the closing phase. Even within Russia, coverage is not even, with two federal districts—North Caucasian and Southern—contributing no distinct entries to the prevalence analysis. Furthermore, most research is concentrated in major regional centers, such as Moscow, St. Petersburg, and Novosibirsk, while other cities within the respective federal districts are represented by one or two reports, if at all. Although Russia accounts for most records in the dataset, this predominance largely reflects its population size of 143.6 million, which is roughly a half of the total FSU population. Therefore, its increased representation is expected and likely reflects both this demographic distribution and a historically higher volume of published epidemiological activity. It should be noted that our search protocol did not extend to national electronic databases published in the respective national languages, which, while unlikely to substantially alter the pooled estimates given their limited volume and topical relevance, represents a formal gap in comprehensive coverage. Additionally, our search of English-language literature was limited to the PubMed database. This may have resulted in the omission of relevant studies. Expanded surveillance in other FSU countries remains essential to achieve truly balanced regional coverage. Such efforts would refine estimates of the region’s disease burden and inform the development of targeted management and prevention strategies.

Temporal coverage is similarly imbalanced: although the review spans almost three decades, more than 95% of available data were generated after 2010. The scarcity of studies from the early 2000s introduces uncertainty and likely inflates pooled prevalence because those early years are dominated by pediatric LRTI cohorts. Representation of clinical presentations other than ARI is also limited, with only 13 studies reporting alternate diagnostic categories. Community-based studies of asymptomatic individuals are effectively absent, with only one such study identified. These limitations constrain the generalizability of the pooled estimates and underscore the need for more geographically and temporally comprehensive RSV surveillance. Future work should aim to expand data collection across under-represented countries, particularly in Central Asia, and improve reporting across diverse clinical settings to provide a more complete and representative view of RSV epidemiology in the region.

## 5. Conclusions

This systematic review and meta-analysis provides the first integrative synthesis of RSV epidemiology across Russia and other countries of the FSU. It outlines broad patterns in RSV prevalence, temporal and seasonal fluctuations, and genetic composition. The highest prevalence was observed in infants and younger children, with rates comparable with those reported globally, and decreasing gradually with age. Meta-regression showed a strong association between clinical presentation and RSV-positivity, with the greatest prevalence observed for LRTI cases, consistent with the established role of RSV in the development of pediatric infections. Genotypic data identified the cosmopolitan clades A.D (ON1), B.D (BA), and their descendants as being dominant in the region since the mid-2010s.

The review identified multiple gaps in the FSU RSV research: (a) most records originate from Russia, with poor representation from the rest of the FSU; (b) limited data are available for senior adults; (c) RSV genomic diversity remains largely unexplored. Nevertheless, the review establishes the region-wide baseline for RSV epidemiology, highlighting its common patterns and providing a solid foundation for future surveillance and comparative work.

The results of the review evidently highlight infants and younger children as the most vulnerable group for RSV infection and its severity onset, which implies that this group is a top priority for efforts focused on further epidemiological research and introduction of novel interventions and vaccines.

## Data Availability

The original contributions presented in this study are included in the article/Supplementary Materials. Further inquiries can be directed to the corresponding author.

## Supplementary Materials

The following supporting information can be downloaded at: https://www.mdpi.com/article/10.3390/v18010126/s1, Supplementary File S1: RSV-related records and extracted sequences; Figure S1: RSV prevalence temporal patterns for Russian federal districts, Baltic States, States of Central Asia and Transcaucasia. Prevalence was pooled across biennial bins via random-effects meta-analysis (REML estimator with Hartung–Knapp–Sidik–Jonkman correction for confidence intervals); Figure S2: RSV seasonality in low-testing-volume FluNet entries, purple bars denote total number of tests performed, blue lines and dots denote RSV prevalence; Figure S3: RSV clades yearly distribution; Table S1: Queries used to retrieve records; Table S2: Inclusion and exclusion criteria used for study selection; Table S3: RSV prevalence summary table; Table S4: Records reporting genotyping data and retrieved RSV clade counts; Table S5: Data duplicates; Table S6: Quality checklist for prevalence studies critical appraisal; Table S7: RSV monthly prevalence in Russia, Kazakhstan, and other FSU countries.

## Appendix A

**Table A1:** Studies included in the systematic review and meta-analysis.

First Author (English)	Year	Date	Broad Age Group	Setting	Location	Clinical Outcomes	Reference
Yatsyshina	2025	October 2021–May 2022, October 2023–August 2024	Mixed	In and out	Russia: 89 regions	ARI	[32]
Sivets	2019	October 2010–October 2018	<18 y	In	Belarus	ARI	[59]
Kurskaya	2022	November 2019–March 2020	Mixed	In	Russia: Yakutsk	ARI	[60]
Saroyan	2022	October 2017–April 2022	<18 y	In	Russia: Novosibirsk	SARI	[61]
Kurskaya	2023	October2022–May 2023	<18 y	In	Russia: Novosibirsk	ARI	[62]
Baimukhametova	2021	January–May 2019	Mixed	In	Kazakhstan	ARI	[63]
Baimukhametova	2019	January–May 2019	Mixed	In	Kazakhstan	ARI	[64]
Yatsyshina	2016	2004–2014	<18 y	In	Russia: Moscow	Pneumonia, conditionally healthy	[65]
Sintsova	2017	2013–2016	Mixed	In	Russia: St. Petersburg	ARI	[66]
Kotlova	2019	October 2017–September 2018	<18 y	In	Russia: Voronezh	ARI	[67]
Kaluzhskikh	2018	2016–2017	<18 y	In	Russia: Kirov	ARI	[68]
Kozhevnikova	2007	November 2001–February 2002	<18 y	In	Russia: Moscow	ARI	[69]
Babachenko	2018	March 2015–February 2017, March 2015–October 2016, March 2015–March 2016, September 2015–November 2016	<18 y	In	Russia: St. Petersburg, Arkhangelsk, Kazan, Saratov	ARI	[70]
Rovnii	2013	April 2011–March 2012	<18 y	In	Russia: St. Petersburg	LRTI	[34]
Gladkikh	2018	September 2014–March 2017	<18 y	In	Russia: Khabarovsk	ARI	[71]
Krasnova	2021	2018–2019	<18 y	In	Russia: Novosibirsk	LRTI	[72]
Reznik	2023	2020–2022	Mixed	In and out	Russia: Khabarovsk	ARI, Pneumonia	[73]
Tolstova	2024	November 2022–February 2023, November 2023–March 2024	<18 y	In	Russia: Moscow	ARI	[74]
Lvov	2020	October 2019–June 2020	Mixed	In	Russia: European Region, Ural and Siberia, Far East	ARI	[37]
Trotsenko	2020	October 2017–April 2019	Mixed	In and out	Russia:Sakha Republic, Primorye, Amur, Sakhalin, Khabarovsk	ARI	[75]
Sominina	2015	January–June 2013	Mixed	In	Russia: St. Petersburg	ARI	[76]
Brusnigina	2025	2021–2024	<18 y	In	Russia: Nizhny Novgorod	Pneumonia	[77]
Lvov	2014	September 2013–July 2014	Mixed	In	Russia: St. Petersburg	ARI	[78]
Bochkareva	2022	2018–2019	<18 y	In	Russia: Zabaykalsk	LRTI, Conditionally healthy	[79]
Yatsyshina	2021	August–October 2020	Mixed	Community	Russia: 26 unspecified regions	Asymptomatic	[31]
Bochkareva	2018	January 2016–December 2017	<18 y	In	Russia: Chita	ARI	[80]
Bogdanova	2018	March 2015–October 2016	<18 y	In	Russia: Arkhangelsk	LRTI	[81]
Semeonova	2016	October–December 2009, 2010–2013, October2013–May 2014	Mixed	In and out	Belarus: Grodno	ARI	[82]
Brusnigina	2021	2018–2020	<18 y	In	Russia: Nizhny Novgorod	LRTI	[83]
Popkova	2013	2011 y	<18 y	In	Russia: Orenburg	ARI	[84]
Kim	2012	January–October 2008, October–December 2011	<18 y	In	Russia: Moscow	Pneumonia	[85]
Malova	2016	2011–2014	Mixed	In	Russia: Penza	ARI	[86]
Ksenafontov	2021	October 2015–April 2021	Mixed	In and out	Russia: St. Petersburg	ARI	[87]
Smorodintseva	2022	October 2013–September 2019	Mixed	In and out	Russia: 11 regions	ARI	[33]
Sergeeva	2013	October 2011–April 2012	Mixed	In and out	Russia: Novosibirsk	ARI	[88]
Tsygankov	2023	January 2021–January 2023	<18 y	In	Russia: Moscow	LRTI	[89]
Deulin	2023	November 2022–February 2023	<18 y	Out	Russia: Berdsk (Novosibirsk region)	ARI	[90]
Reznik	2010	January–March 2010	Mixed	In and out	Russia: Khabarovsk	ARI	[91]
Khaliullina	2018	2016	<18 y	In	Russia: Kazan	SARI	[92]
Pisareva	2018	October 2012–May 2016	Mixed	In	Russia: St. Petersburg	ARI	[93]
Patrusheva	2012	October 2008–April 2009, October 2009–April 2010, October 2010–April 2011, October–December 2011,	<18 y	In	Russia: Moscow	LRTI	[94]
Chudakova	2022	2021	<18 y	In	Russia: Saratov	ARI	[95]
Reznik	2011	June–September 2011, April–November 2010	Mixed	In and out	Russia: Khabarovsk	ARI	[96]
Puzyreva	2025	2021–2023	<18 y	In	Russia: Omsk	ARI	[97]
Caini	2022	January–April 2014, October 2014–April 2019	Mixed	In and out	Russia: Kaliningrad, St. Petersburg, Lipetsk, Chita, Vladivostok, Khabarovsk	ARI	[35]
Sominina	2021	December 2018–May 2019	Mixed	In	Russia: St. Petersburg, Ekaterinburg, Novosibirsk	ARI	[98]
Kampenusa	2024	December 2022–April 2023	Mixed	In and out	Latvia	ARI	[39]
Stacevičienė	2024	October 2021–April 2023	<18 y	In	Lithuania: Vilnus	ARI	[99]
Khomenko	2021	October 2018–March 2020	<18 y	In	Ukraine: Kyiv	ARI	[100]
Tatochenko	2010	September 2008–April 2009	<18 y	In	Russia: Moscow, St. Petersburg, Tomsk	LRTI	[101]
Kurskaya	2024	November 2019–April 2022	<18 y	In	Russia: Novosibirsk	ARI	[102]
Balmaks	2014	July 2009–June 2012	<18 y	In	Latvia	ARI	[103]
Balmaks	2013	May 2009–July 2010	<18 y	In	Latvia	LRTI	[104]
Ghazaryan	2019	November 2013–April 2014, November 2013–April 2014	<18 y	In	Armenia: Yerevan	ARI	[30]
Chkhaidze	2006	1997–2003	<18 y	In	Georgia	ARI	[28]
Chakhunashvili	2018	September 2015–March 2017	Mixed	In	Georgia: Tbilisi, Kutaisi	SARI	[105]
Klivleyeva	2023	2018–2022	Mixed	In and out	Kazakhstan	ARI	[106]
Kurskaya	2018	October 2013–April 2017	<18 y	In	Russia: Novosibirsk	ARI	[107]
Prokopyeva	2023	October 2017–March 2018	<18 y	In	Russia: Novosibirsk	ARI	[108]
Smorodintsev Research Institute of Influenza	2022–2025	2022–2025	Mixed	In and out	Russia	ARI	[20]
“National Center for Public Health” of the Ministry of Health of The Republic of Kazakhstan	2020–2025	2020–2025	Mixed	In and out	Kazakhstan	ARI	[21]
Krivitskaya	2017	2013–2016	<18 y	In	Russia: St. Petersburg	ARI	[109]
Chernysheva	2025	2023–2024	<18 y	In	Russia: Sverdlovsk	ARI	[110]
Krivitskaya	2021	January–April 2014	<18 y	In	Russia: St. Petersburg	ARI	[111]
Sominina	2025	2018–2024	<18 y	In	Russia: St. Petersburg, Ekaterinburg, Novosibirsk,	ARI	[112]
Meslé	2023	2020–2022	Mixed	In and out	Armenia, Belarus, Estonia, Georgia, Moldova, Russia, Ukraine, Uzbekistan, Turkmenistan	ARI	[29]
WHO FluNet	2020–2025	2020–2025	Mixed	In and out	Armenia, Georgia, Moldova, Uzbekistan, Turkmenistan, Kyrgyzstan, Ukraine, Belarus, Estonia, Latvia, Lithuania	ARI	[22]
Sominina	2021	2015–2021	Mixed	In and out	Russia	ARI	[36]
