# Supplementary material for: Respiratory Syncytial Virus Prevalence and Genotypic Distribution in the Countries of the Former Soviet Union: A Systematic Review and Meta-Analysis
Source: Viruses. 2026 Jan 19;18(1):126. doi: 10.3390/v18010126 (PMC12846556; doi:10.3390/v18010126)
Supplement: Supplementary file 1 [file viruses-18-00126-s001.zip › Supplementary File 1.pdf]

Supplementary File 1. RSV-related records and extracted sequences

VGARus accessed data: crie067021:crie067028; crie067591; crie067594; crie067597; crie067599:crie067601; crie068182:crie068185; crie068187:crie068189; crie070125:crie070130; crie070480:crie070481; crie070492:crie070515; crie071263:crie071272; crie071293; crie073823; crie076472:crie076474; crie077032:crie077042; crie077957:crie077963; crie077970:crie077973; crie078532:crie078568; vekt001891:vekt001894; vekt001944:vekt001954; vekt001993; vekt001995:vekt002024; vekt002026:vekt002034; vekt002039:vekt002044; vekt002062:vekt002071; vekt002423:vekt002427; vekt002432; vekt002796:vekt002824

GenBank accessed sequences: MF145160:MF145161; MF145166:MF145168; MF145172:MF145189; MH142221:MH142238; MK386440:MK386445; MH142221:MH142238; MK386440:MK386445; JF979145:JF979157; MK481079; MK534510:MK534512
