# Supplementary figures and images for: Respiratory Syncytial Virus Prevalence and Genotypic Distribution in the Countries of the Former Soviet Union: A Systematic Review and Meta-Analysis
Source: Viruses. 2026 Jan 19;18(1):126. doi: 10.3390/v18010126 (PMC12846556; doi:10.3390/v18010126)

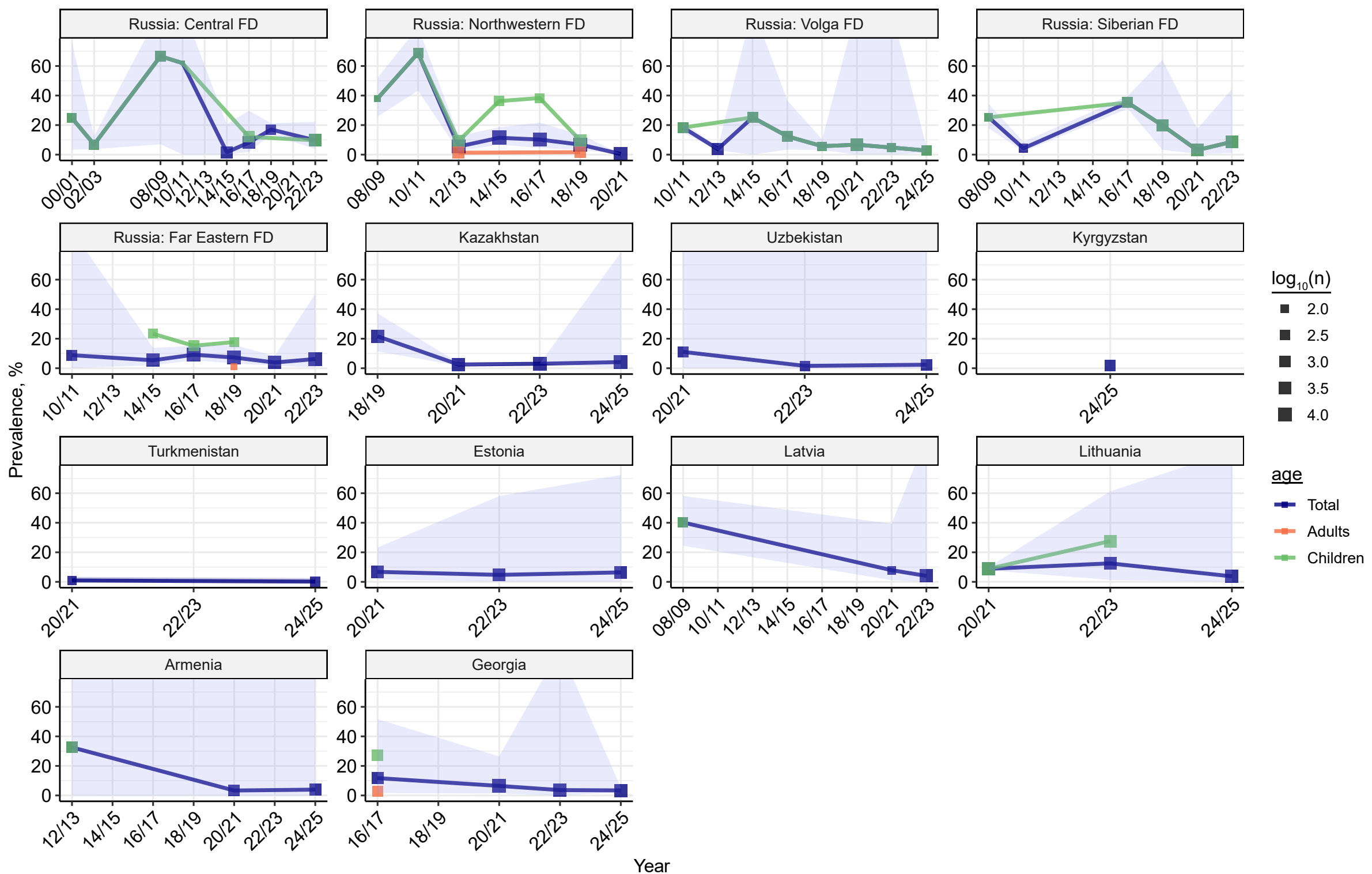

Supplement: Supplementary file 1 [file viruses-18-00126-s001.zip › Figure S1.pdf]

FluNet: Uzbekistan

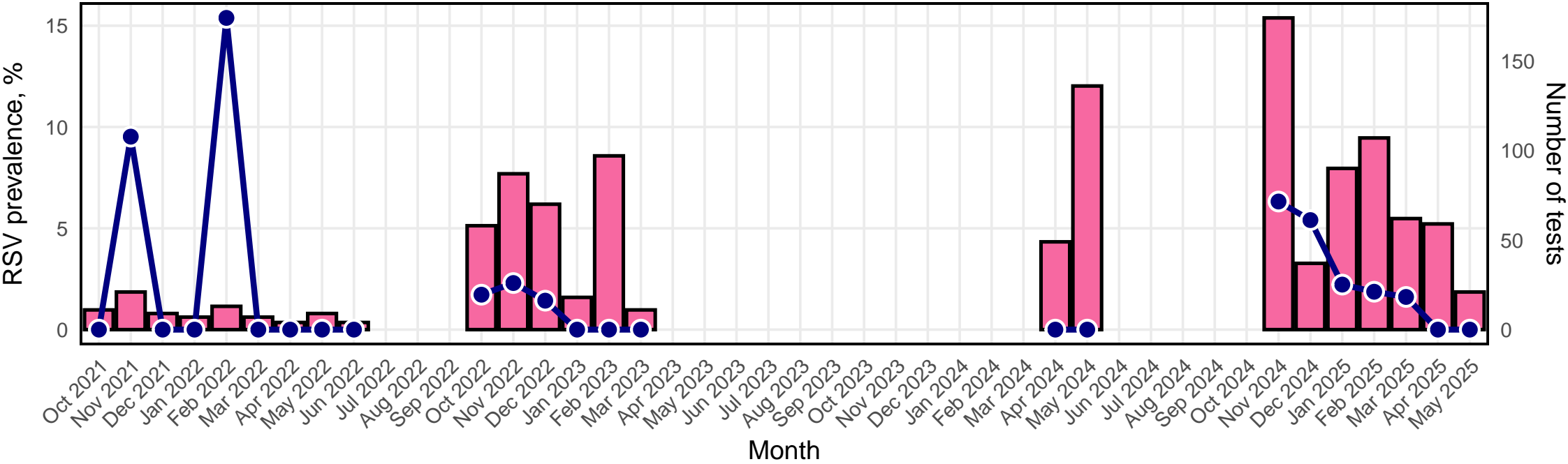

FluNet: Kyrgyzstan

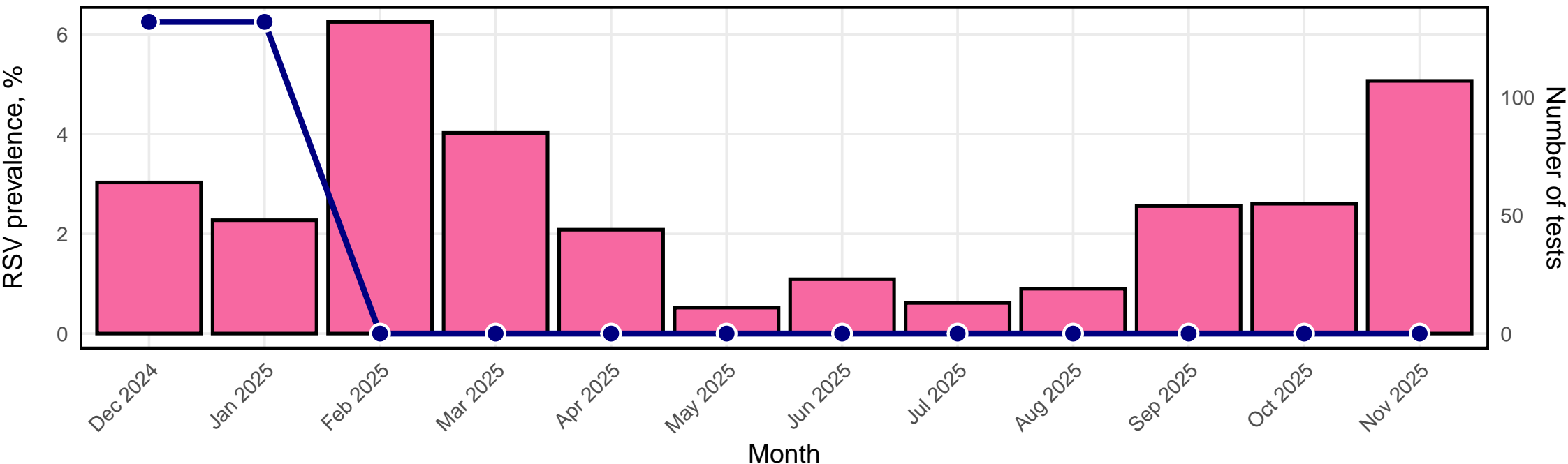

FluNet: Turkmenistan

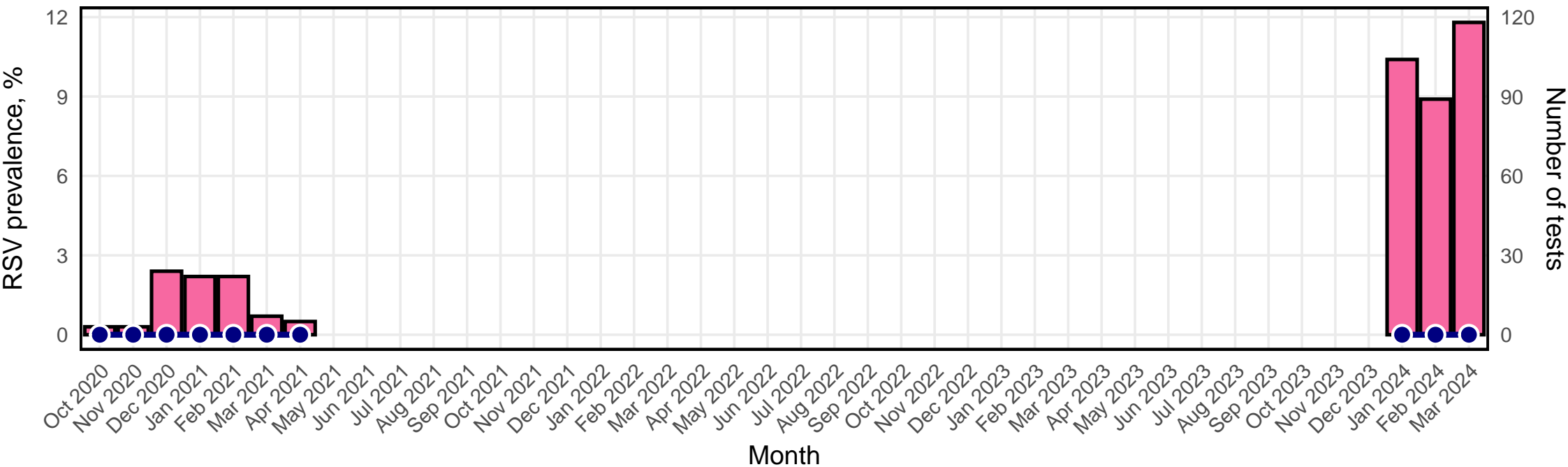

FluNet: Latvia

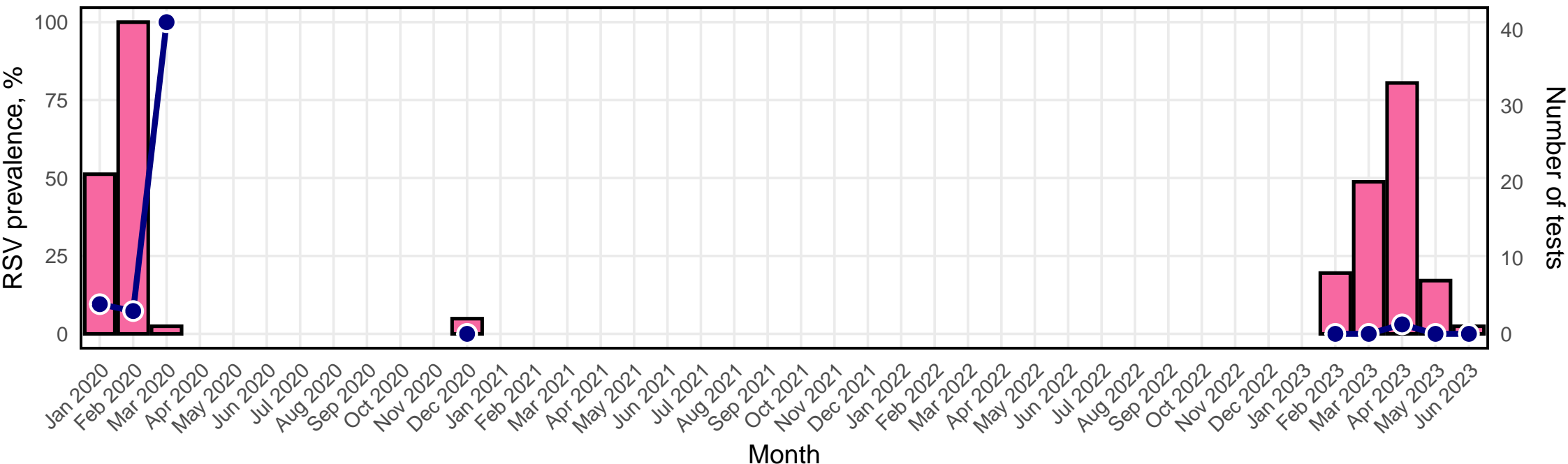

Supplement: Supplementary file 1 [file viruses-18-00126-s001.zip › Figure S2.pdf]

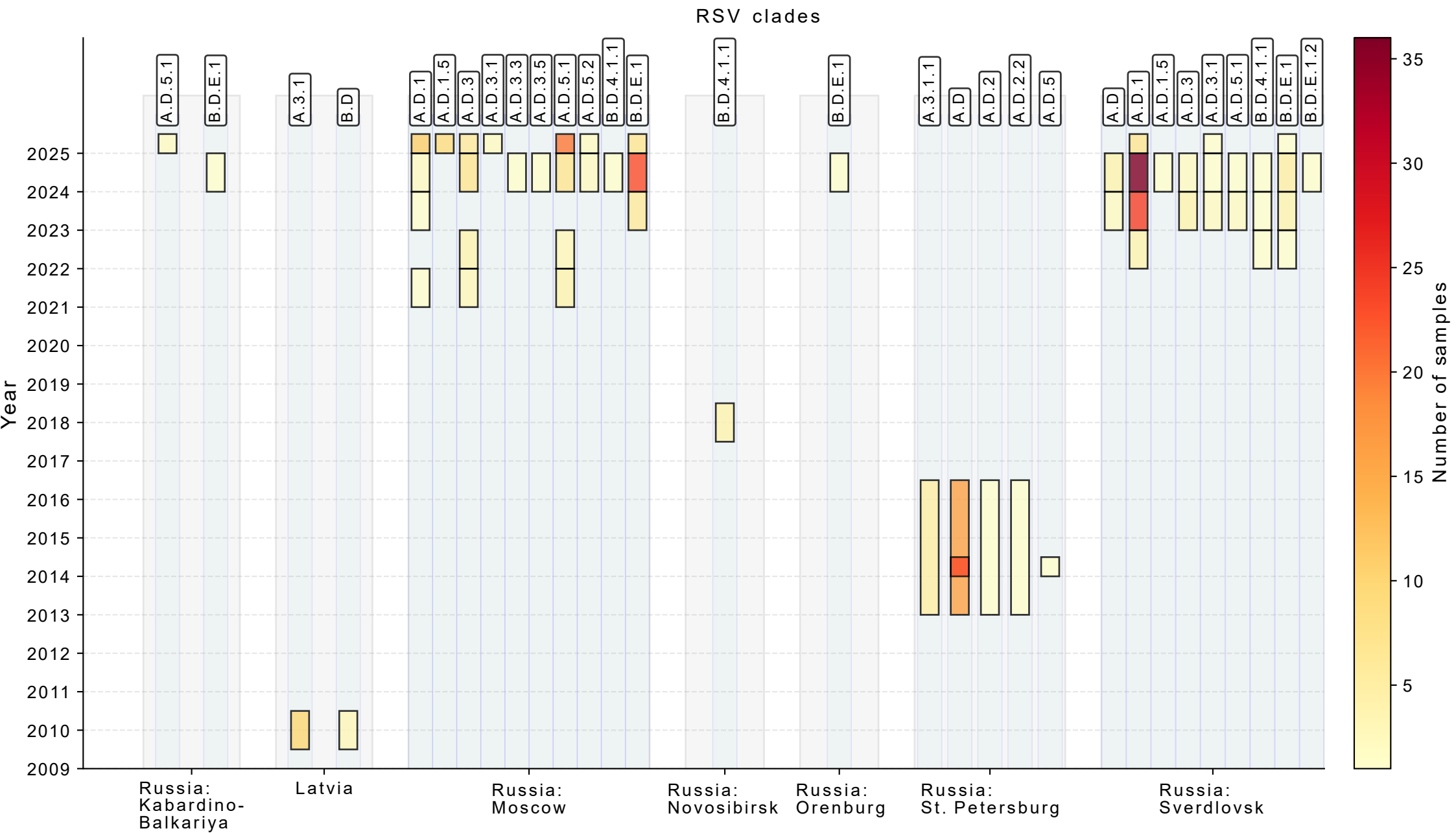

Supplement: Supplementary file 1 [file viruses-18-00126-s001.zip › Figure S3.pdf]
